# Prognosis of papillary thyroid cancer in patients with Graves’ disease: a propensity score-matched analysis

**DOI:** 10.1186/s12957-020-02044-x

**Published:** 2020-10-13

**Authors:** Hyungju Kwon, Byung-In Moon

**Affiliations:** Department of Surgery, Ewha Womans University Medical Center, 1071 Anyangcheon-ro, Yangcheon-Gu, Seoul, 07985 South Korea

**Keywords:** Graves’ disease, Papillary thyroid carcinoma, Recurrence, Prognosis

## Abstract

**Background:**

Patients with Graves’ disease (GD) are at a 2.5 times higher risk of developing thyroid cancer than the general population. Previous studies reported conflicting results about the prognosis of thyroid cancer concomitant with GD. This study aimed to investigate the effect of GD to the recurrence rates of papillary thyroid carcinoma (PTC).

**Methods:**

We reviewed 3628 patients who underwent total thyroidectomy for PTC at the Ewha Womans University Medical Center from January 2006 to June 2014. Of those, 114 patients had non-occult PTC with concomitant GD. To reduce potential confounding effects and selection bias, we conducted 1:5 propensity score matching and analyzed the recurrence-free survival.

**Results:**

Thyroid cancer in patients with GD showed lower rate of lymphatic invasion (1.8% vs. 6.7%; *p* = 0.037), microscopic resection margin involvement (0.9% vs. 5.8%; *p* = 0.024), and lymph node metastasis (29.8% vs. 37.3%; *p* = 0.001) than in patients without GD, respectively. During the median follow-up of 94.1 months, recurrence occurred in one patient (0.9%) with GD. After propensity score matching for adjusting clinicopathological features, 5-year recurrence-free survival was comparable between patients with GD and euthyroid patients (100% vs. 98.4%, *p* = 0.572). Both tumor size [hazard ratio (HR) 1.585, *p* < 0.001] and lymph node metastasis (HR for N1a 3.067, *p* = 0.024; HR for N1b 15.65, *p* < 0.001) were predictive factors for recurrence-free survival, while GD was not associated with the recurrence.

**Conclusions:**

Our data suggest that GD does not affect the prognosis of PTC. Thyroid cancer in patients with GD is not more aggressive than in euthyroid patients.

## Background

Graves’ disease (GD) is an autoimmune thyroid disease and is considered the most common cause of hyperthyroidism [1]. The prevalence of GD is about 0.5% in the general population, with a lifetime risk of 0.5% for men and 3% for women. The central mechanism of GD is an activation of thyroid-stimulating autoantibodies (TSAbs), which provoke the overproduction of thyroid hormone [2]. As the binding of TSAbs to thyrotropin receptor promotes tumor formation and angiogenesis, GD can be associated with an increased risk of thyroid cancer [3, 4]. TSAbs also upregulate various growth factors and enhance tumor invasiveness [5, 6]. Autoimmunity of GD and altered host immune tolerance further increases the risk of thyroid cancer [7, 8].

Thyroid cancer in patients with GD was reported with incidences varying from 2.3 to 21.1% [9–12]. A meta-analysis indicated that the incidence of differentiated thyroid carcinoma was roughly 2.5 times higher in patients with GD than in the general population [4]. In recent two nationwide cohort studies, the hazard ratio for developing thyroid cancer might be up to 12-fold higher for patients with GD than the general population [11, 12]. Over 85% of thyroid cancer in patients with GD were papillary thyroid carcinoma (PTC), although other histologic types including follicular, medullary, and anaplastic carcinoma could be found in those studies. The higher prevalence of thyroid cancer raised interests about the prognosis in patients with GD.

There is an ongoing debate about the prognosis of thyroid cancer associated with GD. Some previous studies indicated that recurrence and disease-specific mortality of thyroid cancer were higher in patients with GD than in euthyroid patients [13–15]. Pellegriti et al. reported that the cumulative risk for recurrent thyroid cancer was approximately three times higher in patients with GD than in euthyroid patients [13]. On the contrary, other studies demonstrated that thyroid cancer in patients with GD had an excellent prognosis and longer disease-free survival [16, 17]. These inconsistent results are because of, at least in part, limited number of patients or unadjusted clinicopathological characteristics. Furthermore, some studies included occult thyroid cancer after thyroidectomy, which had little impact to the recurrence.

This study therefore investigated the recurrence rates of non-occult PTC in patients with GD and compared them with the rates in matched euthyroid patients.

## Methods

### Patients

This retrospective cohort study was approved by the institutional review board (IRB No. 2019-10-039-001), and the need for written informed consent was waived. Between January 2006 and June 2014, 3628 patients with papillary thyroid carcinoma underwent total thyroidectomy. In all patients, thyroid nodules were detected prior to surgery, and fine needle aspiration was preoperatively performed for suspicious nodules. We excluded cases of occult thyroid cancer, which was occasionally found at histologic exam after surgery. Of those 3628 patients, 114 non-occult cases with concomitant GD were included in the present study. All patients underwent neck ultrasonography (USG) and computed tomography preoperatively to evaluate tumor location and cervical lymph node (LN) metastasis. Patients with suspicious LN enlargement performed therapeutic LN dissection in addition to total thyroidectomy.

Data pertaining to patient age, sex, body mass index, and pathological features, including tumor size, extrathyroidal extension (ETE), resection margin involvement, and LN metastasis were collected. Follow-up duration and recurrence status were also recorded. The primary outcome measure was the rate of recurrences.

### Postoperative management and follow-up

All patients underwent thyroid-stimulating hormone suppression therapy. Radioactive iodine treatment was considered in patients with high risk for recurrence (incomplete tumor resection, gross ETE, or distant metastases). Follow-up evaluations, including physical examination, neck ultrasound, serum thyroglobulin, and thyroglobulin antibodies, were performed at intervals of 6–12 months.

### Statistical analysis

To reduce potential confounding effects and selection bias, we conducted 1:5 propensity score matching. We selected the following eight factors which could affect recurrence: age, sex, tumor size, ETE, lymphatic invasion, vascular invasion, resection margin involvement, and LN metastasis.

SPSS Statistics for Windows, version 22.0 (IBM Corp, Armonk, NY, USA) was used for data analysis. Continuous data were compared using Student’s *t* test. Comparisons of categorical data were performed with the chi-squared test. Recurrence-free survival (RFS) was assessed by using Kaplan-Meier plots and the log-rank test. Cox proportional hazards regression model was used to evaluate the relationship between recurrence and prognostic factors. Statistical significance was determined using a *p* value of < 0.05 as the threshold.

## Results

### Characteristics of the patients with GD

The baseline characteristics of the patients are summarized in Table 1. The median follow-up period of patients with GD was 94.1 months [interquartile range (IQR), 69.3–118.8 months]. Thyroid cancer showed lower rate of lymphatic invasion (1.8% vs. 6.7%; *p* = 0.037) and microscopic resection margin involvement (0.9% vs. 5.8%; *p* = 0.024) in patients with GD than in patients without GD. Patient with GD also showed lower rate of LN metastasis (29.8% vs. 37.3%; *p* = 0.001). Distant metastasis was not found in all patients. There was no significant difference in sex, age, tumor size, presence of ETE, and AJCC 7th TNM classification.

**Table 1:** Comparison of clinicopathological features between patients with and without Graves’ disease

Characteristics	PTC with GD (*n* = 114)	PTC without GD (*n* = 3514)	*p* value
Age (years)	46.3 ± 12.9	45.8 ± 12.2	0.641
Female sex	99 (86.8%)	2916 (83.0%)	0.279
Body mass index (kg/m^2^)	23.8 ± 3.1	23.5 ± 3.2	0.396
Preoperative FNA results			0.197
Benign	0 (0.0%)	0 (0.0%)	
Atypia	7 (6.1%)	62 (1.8%)	
Follicular neoplasm	0 (0.0%)	166 (4.7%)	
Suspicious malignancy	30 (26.3%)	841 (23.9%)	
Malignancy	77 (67.5%)	2344 (66.7%)	
Pathologic features			
Tumor size (cm)	0.8 ± 0.5	0.9 ± 0.7	0.057
Extrathyroidal extension			0.317
No	59 (51.8%)	1663 (47.3%)	
Microscopic	51 (44.7%)	1611 (45.8%)	
Gross	4 (3.5%)	240 (6.8%)	
Lymphatic invasion	2 (1.8%)	234 (6.7%)	0.037
Vascular invasion	0 (0.0%)	43 (1.2%)	0.235
Margin involvement	1 (0.9%)	205 (5.8%)	0.024
Number of retrieved LNs	6.0 ± 5.8	6.7 ± 8.1	0.208
LN metastasis			0.001
N0	80 (70.2%)	2203 (62.7%)	
N1a	34 (29.8%)	950 (27.0%)	
N1b	0 (0.0%)	361 (10.3%)	
^131^I remnant ablation	43 (37.7%)	1545 (44.0%)	0.186
^131^I dose (mCi)	47.2 ± 32.8	41.8 ± 35.8	0.331
Follow-up (months)	96.8 ± 29.5	96.5 ± 31.0	0.894
Recurrence			0.487
Regional recurrence	1 (0.9%)	62 (2.5%)	
Distant metastasis	0 (0.0%)	5 (0.2%)	

Data presented as mean and standard deviation if not noted otherwise. Categorical data were compared using the chi-squared test. Data derived from continuous variables of different groups were compared by Student’s *t* test

*PTC* papillary thyroid carcinoma, *GD* Graves’ disease, *FNA* fine needle aspiration, *LN* lymph node

### Comparison of the recurrences rates

**Fig. 1:**
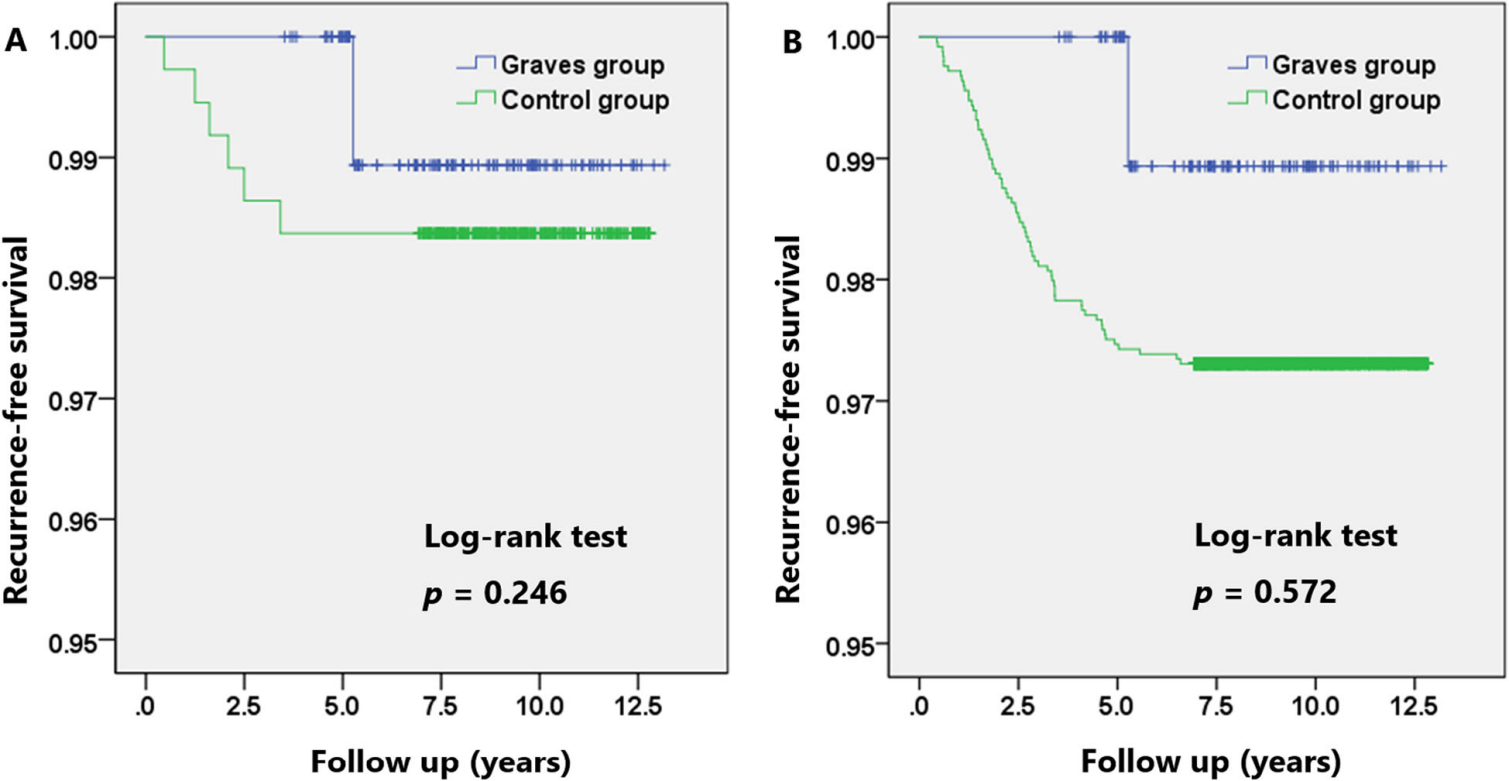
Recurrence-free survival for patients with or without Graves’ disease. **a** Recurrence-free survival before matching. **b** Recurrence-free survival after 1:5 matching

Recurrence was found in one patient (0.9%) in GD group, and 67 patients (2.7%) without GD developed recurrence (*p* = 0.487) including five patients with distant metastasis, respectively. The recurrence-free survival (RFS) showed no difference; the 5-year RFS for patients with GD was comparable with those without GD (100.0% vs. 97.5%, *p* = 0.246; Fig. 1a). To control the differences of baseline characteristics, patients with GD were 1:5 propensity matched to yield 114 matched pairs of 684 patients. Table 2 showed the clinicopathological comparison between the GD group versus the matched patients in euthyroid state without GD. The matched cohorts did not differ in terms of pathological features including tumor size, ETE, lymphatic invasion, vascular invasion, resection margin involvement, and LN metastasis. After adjustment of possible confounders, the overall recurrence rates did not significantly differ between groups (0.9% in GD group vs. 1.6% in matched control group; *p* = 0.557), during the median follow-up of 94.1 months (interquartile ratio 69.3–118.8 months, *p* = 0.894 between groups). The 5-year RFS was also comparable between the matched groups (100.0% vs. 98.4%, *p* = 0.572; Fig. 1b).

**Table 2:** Patient demographics and pathologic features after propensity-score matching

Characteristics	PTC with GD (*n* = 114)	PTC without GD (*n* = 570)	*p* value
Age (years)	46.3 ± 12.9	46.3 ± 12.1	0.960
Female sex	99 (86.8%)	486 (85.3%)	0.662
Body mass index (kg/m^2^)	23.8 ± 3.1	23.5 ± 3.1	0.422
Preoperative FNA results			0.142
Benign	0 (0.0%)	9 (1.6%)	
Atypia	7 (6.1%)	26 (4.6%)	
Follicular neoplasm	0 (0.0%)	22 (3.9%)	
Suspicious malignancy	30 (26.3%)	139 (24.4%)	
Malignancy	77 (67.5%)	374 (65.6%)	
Pathologic features			
Tumor size (cm)	0.8 ± 0.5	0.7 ± 0.4	0.386
Extrathyroidal extension			0.959
No	59 (51.8%)	290 (50.9%)	
Microscopic	51 (44.7%)	257 (45.1%)	
Gross	4 (3.5%)	23 (4.0%)	
Lymphatic invasion	2 (1.8%)	10 (1.8%)	1.000
Vascular invasion	0 (0.0%)	0 (0.0%)	NA
Margin involvement	1 (0.9%)	5 (0.9%)	1.000
LN metastasis			0.648
N0	80 (70.2%)	412 (72.3%)	
N1a	34 (29.8%)	158 (27.7%)	
N1b	0 (0.0%)	0 (0.0%)	
^131^I remnant ablation	43 (37.7%)	236 (41.4%)	0.465
^131^I dose (mCi)	47.2 ± 32.8	43.9 ± 42.6	0.636
Follow-up (months)	96.8 ± 29.5	94.0 ± 31.2	0.894
Recurrence			0.557
Regional recurrence	1 (0.9%)	6 (1.6%)	
Distant metastasis	0 (0.0%)	0 (0.0%)	

Data presented as mean and standard deviation if not noted otherwise. Categorical data were compared using the chi-squared test. Data derived from continuous variables of different groups were compared by Student’s *t* test

*PTC* papillary thyroid carcinoma, *GD* Graves’ disease, *FNA* fine needle aspiration, *LN* lymph node, *NA* not applicable

### Predictive factors of recurrence

Univariate Cox-proportional hazard analysis showed that ETE (hazard ratio [HR] for microscopic ETE 3.146, *p* = 0.001; HR for gross ETE 10.22, *p* < 0.001), lymphatic invasion (HR 5.520, *p* < 0.001), vascular invasion (HR 4.990, *p* = 0.002), resection margin involvement (HR 2.238, *p* = 0.019), LN metastasis (HR for N1a 3.331, *p* = 0.003; HR for N1b 22.76, *p* < 0.001), and ^131^I remnant ablation (HR 4.858, *p* < 0.001) were significantly associated with the risk of recurrence (Table 3). Multivariate analysis showed that both tumor size (HR 1.585, *p* < 0.001) and LN metastasis (HR for N1a 3.067, *p* = 0.023; HR for N1b 15.65, *p* < 0.001) were independently associated with recurrence. Both univariate and multivariate analyses showed that GD was not a predictive factor of the recurrence of thyroid cancer.

**Table 3:** Predictive factors for recurrence-free survival

Covariates	Univariate analysis		Multivariate analysis	
	HR (95% CI)	*p* value	HR (95% CI)	*p* value
Clinical features				
Graves’ disease	0.329 (0.046–2.371)	0.270	1.299 (0.170–9.941)	0.801
Age at surgery (years)	0.981 (0.962–1.001)	0.068	0.983 (0.964–1.001)	0.067
Male sex	1.092 (0.585–2.037)	0.782	0.669 (0.350–1.277)	0.223
Pathological features				
Tumor size	2.132 (1.822–2.496)	< 0.001	1.585 (1.245–2.018)	< 0.001
Extrathyroidal extension				
Microscopic	3.146 (1.644–6.021)	0.001	1.674 (0.814–3.441)	0.161
Gross	10.22 (4.920–21.21)	< 0.001	2.030 (0.931–4.958)	0.120
Lymphatic invasion	5.520 (3.112–9.793)	< 0.001	1.440 (0.763–2.718)	0.261
Vascular invasion	4.990 (1.817–13.70)	0.002	1.536 (0.540–4.371)	0.421
Margin involvement	2.238 (1.144–4.378)	0.019	0.881 (0.400–1.642)	0.560
LN metastasis				
N1a	3.331 (1.192–7.435)	0.003	3.067 (1.160–8.111)	0.024
N1b	22.76 (11.76–44.08)	< 0.001	15.65 (6.404–38.25)	< 0.001
^131^I remnant ablation	4.858 (2.323–10.16)	< 0.001	0.622 (0.223–1.739)	0.366

Data presented as mean and standard deviation if not noted otherwise. Cox proportional hazard model was used for univariate and multivariate analysis for recurrence-free survival

*HR* hazard ratio, *CI* confidence interval, *LN* lymph node

## Discussion

This study demonstrated that GD did not affect the prognosis of the patients with PTC. From the 1990s, GD has been believed to be associated with aggressive thyroid cancer [18, 19]. High serum levels of TSAbs from GD can stimulate growth and metastasis of thyroid cancer [20–22]. Chronic abnormal stimulation by TSAbs may lead to more aggressiveness of thyroid cancer in patients with GD [22]. However, recent studies indicated that humoral immune response triggered by GD was protective against thyroid cancer [23]. Increased concentration of activated NK cells or M1 macrophage provided tumor-protective immunity, which resulted in less aggressive thyroid cancer. Yoshioka et al. further indicated that immunologic remission of TSAbs could be obtained by surgery, which eliminated the deleterious effect of TSAbs [24].

In the present study, 5-year RFS of PTC in patients with GD was comparable, at least not bad, to that in euthyroid patients (100% vs. 99.6%, *p* = 0.516). Kikuchi et al. suggested two possible mechanisms for the good prognosis of thyroid cancer with GD [16]. First, the size of thyroid cancer was relatively small due to thyroid hypertrophy, which would make it difficult to invade thyroid capsule or adjacent organs. The other mechanism was that attentive surgical procedure in Graves’ disease led to lesser remnant thyroid tissue. We also found that patients with GD showed lower rate of margin involvement (0.9% vs 5.8%, *p* = 0.024) and comparable rate of ETE (48.2% vs 52.7%, *p* = 0.317) to those without GD, respectively. A lower rate of LN metastasis (29.8% vs 37.3%, *p* = 0.001) in patients with GD further contributed to the good prognosis in our study.

Both tumor size (HR 1.58, 95% CI 1.24–2.01) and LN metastasis (HR 2.48, 95% CI 1.09–5.66) were predictive factors for recurrence in the present study, while GD (HR 1.12, 95% CI 0.15–8.34) was not associated with the recurrence. Tumor size has been recognized as a predictive factor for recurrence in various risk stratification system, including AGES, AMGES, and MACIS score [25]. LN metastasis also has been widely described as a risk factor for recurrence [26–28]. A recent meta-analysis further demonstrated that tumor size over 2 cm (OR 2.69, 95% CI 2.06–3.50) or LN metastasis (OR 3.24, 95% CI 2.61–4.02) significantly increased the risk of recurrence [29]. Recent research from Medas et al. indicated that both LN metastasis and large tumor size were independent predictors of recurrences as well [28]. The result of our study is consistent with previous reports.

There is no consensus or recommended protocol for detection of thyroid cancer in patients with GD, although GD is associated with higher risk of PTC [30]. Thyroid USG can identify more thyroid nodules or cancers in patients with GD, compared with palpation or radioactive iodine scintigraphy [30]. Routine USG for screening could result in the higher detection rate of small PTCs [31]. Some studies, therefore, illustrated that patients with GD tended to have microcarcinomas, which had little impact on recurrence [32]. Conversely, other researchers emphasized that patients with GD needed early detection and aggressive treatment of thyroid cancer [13]. In my institution, annual thyroid USG was recommended to all patients with GD. Annual USG may facilitate early diagnosis of PTC, which can further decrease the risk of recurrence.

This study has some limitations. First, all patients in the GD group underwent thyroidectomy due to concomitant thyroid cancer, although they had well controlled GD. Therefore, the influence of GD on recurrence may have been underestimated. Second, the effect of thyroid-stimulating autoantibodies was not investigated in the present study because there was only one recurrence in patients with GD. Third, we did not evaluate long-term prognosis including mortality. During the follow-up period of 7.8 years, there was no cancer-related death in our cohort. Further validation study in larger cohort is warranted.

## Conclusion

PTC in patients with GD showed excellent prognosis and disease-free survival rates comparable to those of patients with euthyroid states. Thyroid cancer in patients with GD is not more aggressive than in euthyroid patients.

## Supplementary information

**Additional file 1: Supplementary Table 1.** Patient demographics and pathologic features after propensity-score matching using age, sex and tumor size

## Data Availability

The datasets used and/or analyzed during the current study are available from the corresponding author on reasonable request.

## Abbreviations

ETE: Extrathyroidal extension
GD: Graves’ disease
HR: Hazard ratio
IQR: Interquartile range
LN: Lymph node
PTC: Papillary thyroid carcinoma
RFS: Recurrence-free survival
TSAbs: Thyroid-stimulating autoantibodies
