# Supplementary material for: Prognosis of papillary thyroid cancer in patients with Graves’ disease: a propensity score-matched analysis
Source: World J Surg Oncol. 2020 Oct 13;18:266. doi: 10.1186/s12957-020-02044-x (PMC7557089; doi:10.1186/s12957-020-02044-x)
Supplement: Supplementary file 1 — Additional file 1: Supplementary Table 1. Patient demographics and pathologic features after propensity-score matching using age, sex and tumor size [file 12957_2020_2044_MOESM1_ESM.docx]

**Supplementary table 1** Patient demographics and pathologic features after propensity-score matching using age, sex and tumor size

| Characteristics | PTC with GD (n=114) | PTC without GD (n=570) | *p* value |
| --- | --- | --- | --- |
| Gender (male:female) | 15:99 (13.2%:86.8%) | 83:487 (14.6%:85.4%) | 0.696 |
| Age (years) | 46.3 ± 12.9 | 45.6 ± 12.1 | 0.594 |
| Body mass index (kg/m^2^) | 23.8 ± 3.1 | 23.2 ± 3.0 | 0.085 |
| Pathologic features |  |  |  |
| Tumor size (cm) | 0.8 ± 0.5 | 0.8 ± 0.5 | 0.669 |
| Extrathyroidal extension |  |  | 0.650 |
| No | 59 (51.8%) | 285 (50.0%) |  |
| Microscopic | 51 (44.7%) | 253 (44.4%) |  |
| Gross | 4 (3.5%) | 32 (5.6%) |  |
| Lymphatic invasion | 2 (1.8%) | 36 (6.3%) | 0.052 |
| Vascular invasion | 0 (0.0%) | 5 (0.9%) | 0.316 |
| Margin involvement | 1 (0.9%) | 32 (5.6%) | 0.031 |
| LN metastasis |  |  | 0.004 |
| N0 | 80 (70.2%) | 371 (65.1%) |  |
| N1a | 34 (29.8%) | 149 (26.1%) |  |
| N1b | 0 (0.0%) | 50 (8.8%) |  |
| Follow up (months) | 96.8 ± 29.5 | 95.2 ± 30.2 | 0.593 |
| Recurrence | 1 (0.9%) | 6 (1.6%) | 0.557 |

Data presented as mean and standard deviation if not noted otherwise. Categorical data were compared using the chi-squared test. Data derived from continuous variables of different groups were compared by Student T test

*PTC* papillary thyroid carcinoma, *GD* Graves’ disease, *LN* lymph node, *NA* not applicable.
